# Supplementary material for: HIVprotI: an integrated web based platform for prediction and design of HIV proteins inhibitors
Source: J Cheminform. 2018 Mar 9;10:12. doi: 10.1186/s13321-018-0266-y (PMC5845081; doi:10.1186/s13321-018-0266-y)
Supplement: Supplementary file 2 — Additional file 2. Source code of HIVProtI web server. [file 13321_2018_266_MOESM2_ESM.zip › HIVprotI_Source-code/hivprotI/weka-3-6-6/doc/weka/core/xml/package-summary.html]

weka.core.xml


---


|  |  |  |
| --- | --- | --- |
| **Overview** \| **Package** \| Class \| **Tree** \| **Deprecated** \| **Index** \| **Help** \| **Weka's home** | |  |
| **PREV PACKAGE**   **NEXT PACKAGE** | **FRAMES**    **NO FRAMES**     **All Classes** |  |


---

## Package weka.core.xml

| **Class Summary** | |
| --- | --- |
| **KOML** | This class is a helper class for XML serialization using KOML . |
| **MethodHandler** | This class handles relationships between display names of properties (or classes) and Methods that are associated with them. |
| **PropertyHandler** | This class stores information about properties to ignore or properties that are allowed for a certain class. |
| **SerialUIDChanger** | This class enables one to change the UID of a serialized object and therefore not losing the data stored in the binary format. |
| **XMLBasicSerialization** | This serializer contains some read/write methods for common classes that are not beans-conform. |
| **XMLDocument** | This class offers some methods for generating, reading and writing XML documents.  It can only handle UTF-8. |
| **XMLInstances** | XML representation of the Instances class. |
| **XMLOptions** | A class for transforming options listed in XML to a regular WEKA command line string. |
| **XMLSerialization** | With this class objects can be serialized to XML instead into a binary format. |
| **XMLSerializationMethodHandler** | This class handles relationships between display names of properties (or classes) and Methods that are associated with them. |
| **XStream** | This class is a helper class for XML serialization using XStream . |

---


|  |  |  |
| --- | --- | --- |
| **Overview** \| **Package** \| Class \| **Tree** \| **Deprecated** \| **Index** \| **Help** \| **Weka's home** | |  |
| **PREV PACKAGE**   **NEXT PACKAGE** | **FRAMES**    **NO FRAMES**     **All Classes** |  |


---
